# Editorial: Early Life Stress and Depression

**DOI:** 10.3389/fpsyt.2019.00964

**Published:** 2020-01-15

**Authors:** Fushun Wang, Jiongjiong Yang, Fang Pan, James A. Bourgeois, Jason H. Huang

**Affiliations:** ^1^ Institute of Brain and Psychological Science, Sichuan Normal University, Chengdu, China; ^2^ Department of Psychology, Beijing University, Beijing, China; ^3^ Department of Psychology, Shandong University, Jinan, China; ^4^ Department of Psychiatry, Memorial Hospital, Baylor Scott & White Health and Texas A&M University, Temple, TX, United States; ^5^ Department of Neurosurgery, Memorial Hospital, Baylor Scott & White Health and Texas A&M University, Temple, TX, United States

**Keywords:** early life stress, major depression, emotion, monoamine, hypothalamic-pituitary-adrenal axis

Major depressive disorder (MDD) is a leading cause of disability worldwide, affecting 17% of the population, making it one of the most prevalent health-related causes of human suffering. The pathophysiological mechanisms of depression, likely multiple, are far from clear. One widely accepted theory regarding the genesis of depression points to excess external stress beyond the patient’s capacity to cope, especially early life stress. Even though compelling evidence from epigenetic studies and psychopharmacological studies points to dysfunction of hypothalamic-pituitary-adrenal axis (HPA) hormones and the associated central monoamine network, the exact mechanisms of how excessive, or “toxic” early life stress affects the later risk for adult depressive disorders are still not clear.

The mechanisms whereby excess external stressors affect brain function have been the subject of extensive study over the past 60 years. During the early to middle twentieth century, Sigmund Freud posited that maladaptive response to excess early life stress was thereafter embedded in the unconscious mind, leading to adult-onset depressive and other psychiatric disorders. Later, John Bowlby proposed that stressful circumstances contributed to maladaptive attachment patterns between children and parents/other caregivers could predispose to the later development of psychiatric illnesses. Bowlby proposed two broad types of attachments, secure and insecure, with the latter category including anxious and avoidant subtypes. By this model, these insecure, maladaptive attachment styles affect the development of personality characteristics and thus increase the risk of depressive and other psychiatric illnesses in adults.

From a neurosciences perspective, many neuromodulators underlying responses to stress affect brain function, as manifest by psychiatric disorders. Corticotropin-releasing hormone (CRH), the “stress hormone,” CRH release activates the hypothalamic-pituitary-adrenal (HPA) axis, one of the central mechanisms involved in stress. The CRH induces release of ACTH (adrenocorticotropic hormone), which in turn alters function of the neural network thus leading to the behavioral and/or emotional changes. In addition to ACTH, monoamine (including norepinephrine (NE), dopamine (DA) and 5-HT) has been proved to play critical roles in emotional disorders, and has been used as first-line of treatment for MDD for nearly a century. These monoamines are the substrates of many mental disorders, including depression. Indeed, some early life events induce epigenetic changes for many neuromodulator receptors and/or transporters, such as methylation DNA of MAO (monoamine oxidase). These changes may induce dysfunction of monoamines, plausibly related to depressive disorders.

The three monoamine neuromodulators are suggested to be associated with the three core affects (dopamine-reward, serotonin-punishment, norepinephrine-stress), and they work together to make different basic emotions, analogous to the three primary colors. The DA system is involved in reward (leading to joy), NE has been related to the “fight or flight” (leading to fear and anger) responses to stressful events, and the 5-HT system has been related to punishment (leading to sadness) (please refer to our previous publication, such as Front Neurosci 2019 Jun 19; 13:628).

In this special issue, we feature research studies on early life traumatic events with later development of depressive disorders. We are pleased to have collected 42 submissions for this topic; 25 of them have been accepted, including the following:

In the paper titled “Understanding the relation between early-life adversity and depression symptoms: The moderating role of sex and an interleukin-1β gene variant”, McQuaid et al. found that, in the context of early life adversity, genetic variations of IL-1β functioning are related to depressive symptomatology. This study also, more broadly, highlights the importance of considering the confluence of experiential factors (e.g., early life adversity) and personal characteristics (e.g., sex, genetics) in understanding depressive disorders, an approach increasingly recognized in developing personalized treatment approaches to this illness.

In “Long-lasting sex-specific effects based on emotion- and cognition-related behavioral assessment of adult rats after posttraumatic stress disorder from different lengths of maternal separation”, Yang et al. demonstrate that an early mildly stressful experience may be involved in helping the body adapt optimally when faced with additional trauma in adulthood, although mild early life stress might benefit learning and memory among males.

In “Interactive effects of life events and hair cortisol on perceived stress, anxiety and depression symptoms among Chinese adolescents: Testing the differential susceptibility and diathesis-stress models”, Xu et al. examined whether the activity of the hypothalamic pituitary adrenal axis (HPA) can serve as a physiological marker of the differential susceptibility or the diathesis-stress models. The authors explored the interactive effect of life events and hair cortisol on perceived stress, anxiety and depression symptoms among Chinese adolescents. They found that HPA activity might be a physiological marker of the differential susceptibility model for perceived stress and anxiety symptoms, and may serve as a physiological marker of the diathesis-stress model for depression symptoms among Chinese adolescents.

In “State-related alterations of spontaneous neural activity in current and remitted depression revealed by resting-state fMRI”, Cheng et al. explored the altered spontaneous neural activities in major depressive disorder (MDD), using functional magnetic resonance imaging (fMRI). They found abnormal activity in the left middle occipital gyrus, left middle temporal gyrus and anterior right cerebellar lobe to be related to state-specific symptoms of MDD.

In “Altered brain function in drug-naïve major depressive disorder patients with early-life maltreatment: A resting-state fMRI Study”, Xu et al. examined brain function in MDD patients with childhood maltreatment experience using resting-state fMRI (rs-fMRI). They found that MDD patients with history of childhood maltreatment experience demonstrated increased amplitude of low-frequency fluctuation (ALFF) and altered function connection (FC) in prefrontal cortex when compared to MDD patients without childhood maltreatment.

In “Inhibition of GALR1 in PFC alleviates depressive-like behaviors in postpartum depression rat model by upregulating CREB-BNDF and 5-HT levels”, Li et al. examined the role of GAL receptors in postpartum depressive patients. They found that GALR1, rather than GALR2/3, was upregulated with a region-specific pattern in the prefrontal cortex (PFC) of E2 withdrawal induced post-partum depressive disorder model rats. They concluded that GALR1 expression in PFC is involved in depressive-like behaviors.

In “Childhood trauma and sleep among young adults with a history of depression: A daily diary study”, Hamilton et al. evaluated childhood maltreatment and trauma and insomnia symptoms among young adults with depression They found that emotional neglect in early life can predict insomnia symptoms among individuals with a depression history.

In “The interaction of TPH2 and 5-HT2A polymorphisms on major depressive disorder susceptibility in a Chinese Han population: a case-control study”, Yang et al. studied the role of TPH2 and 5-HT2A in MDD. They found that the homeostatic dysfunction of serotonin levels in the brain and their genetic variations may lead to impaired homeostatic regulation of serotonin. This results in abnormal levels of serotonin in the brain, predisposing individuals to MDD.

In “Epistatic interaction between 5-HT1A and VEGF genes polymorphisms in the northern Chinese Han population with major depressive disorder”, Qiao et al. examined the role of the serotonin 1A receptor (5-HT1A) and vascular endothelial growth factor (VEGF) expression in the neurons of the hippocampus. They found that interactions between 5-HT1A and VEGF gene polymorphisms may play a key role in the development of MDD in the Northern Chinese Han population.

In “Dynamic effects of early adolescent stress on depressive-like behaviors and expression of cytokines and JMJD3 in prefrontal cortex and hippocampus of rats”, Wang et al. discovered that the Jumonji domain containing-3 (Jmjd3), which is a histone H3 lysine 27 (H3K27) demethylase, might be involved in the susceptibility to depressive-like behaviors by modulating H3K27me3 and pro-inflammatory cytokine expression in the prefrontal cortex and hippocampus of rats that had been stressed during early adolescence.

In “Alterations of DNA methylation at GDNF gene promoter in the ventral tegmental area of adult depression-like rats induced by maternal deprivation”, Zhang et al. found that up-regulation of DNA methylation at GDNF gene promotor and subsequent down-regulation of GDNF gene expression in the ventral tegmental area were involved in the development of depression-like behaviors in rats experiencing maternal deprivation in early life.

In “Dysfunction of preattentive visual information processing in drug-naïve women, but not men, during the initial episode of major depressive disorder”, Yang et al. examined the gender difference in depressive disorder risk between women and men., They found that women with MDD had dysfunction of visual information processing at pre-attentive stage.

In “Cognitive impairment and endoplasmic reticulum stress induced by repeated short-term sevoflurane exposure in early life of rats”, Shen et al. reported that sevoflurane, one of the most commonly used volatile anesthetics in children, can induce long-term changes in the brain.

In “Earthquake trauma, overgeneral autobiographical memory, and depression among adolescent survivors of the Wenchuan earthquake”, Tian et al. examined the relationship among earthquake trauma, overgeneral autobiographical memory, and depression in adolescent survivors of the Wenchuan earthquake. They found that the adolescents with average and high overgeneral autobiographical memory and experienced more depression than adolescents with no earthquake trauma.

In “Hydrogen sulfide antagonizes chronic restraint stress-induced depressive-like behaviors *via* upregulation of adiponectin”, Tian et al. reported that hydrogen sulfide (H2S), a novel gasotransmitter, antagonizes chronic stress induced depressive-like behaviors in rats, *via* ameliorated synaptic and autophagic dysregulation by upregulation of adiponectin.

In “Minocycline attenuates stress-induced behavioral changes *via* its anti-inflammatory effects in an animal model of post-traumatic stress disorder”, Wang et al. found that minocycline altered anxiety-like behavior and cognitive deficits after post-traumatic stress disorder PTSD.

In “Effects of Xiaoyaosan on the hippocampal gene expression profile in rats subjected to chronic immobilization stress”, Li et al. examined the effects of xiaoyaosan and its anti-stress mechanism in rats subjected to chronic immobilization stress at the whole genome level. They found that inhibition of hippocampal cell growth was the core molecular event of network regulating the transcription of the differentially expressed genes in the model group.

In “Yi-nao-jie-yu prescription exerts a positive effect on neurogenesis by regulating notch signals in the hippocampus of post-stroke depression rats”, Tian et al. examined the therapeutic role of Yi-nao-jie-yu in post-stroke depression (PSD). They found that the drug can relieve depressive behavior in PSD rats, and exerts a positive effect on neurogenesis by dynamically regulating the expression of notch signaling genes.

In “SiNiSan ameliorates the depression-like behavior of maternal separation experienced rats through 5-HT1A receptor/CREB/BDNF pathway”, Cao et al. found that maternal separation in infancy could lead to depression-like behaviors in young and adult rats, and the level of 5-HT1A receptor, p-CREB and BDNF in hippocampus were reduced in these affected rats. The drug SiNiSan treatment significantly up-regulated the expression of the 5-HT1A receptor, p-CREB and BDNF in hippocampus of adult MDD rats/They concluded that SiNiSan treatment may have antidepressant effects on both young and adult MS rats through the BDNF/PKA/CREB pathway.

In “Paeoniflorin ameliorates chronic stress-induced depression-like behaviors and neuronal damages in rats *via* the activation of the ERK-CREB pathway”, Zhong et al. found that paeoniflorin possibly exerted a neuroprotective effect, modulated by the ERK-CREB signaling pathway, inon CUMS-induced hippocampal damage in rats

In “Short-term intrarectal administration of sodium propionate induces antidepressant-like effects in rats exposed to chronic unpredictable mild stress”, Li et al. did a one-week intrarectal administration of sodium propionate which induced antidepressant-like effects. These were correlated with differential rescue of neurotransmitters in the prefrontal cortex. This may have been achieved through the reduction of the catabolism of noradrenaline, tryptophan and dopamine, rather than serotonin.

In “Review of abnormal self-knowledge in major depressive disorder”, Lou et al. reviewed papers on the behavioral and neurological changes of self-knowledge in depressive patients. They found that depressed individuals, as compared with non-depressed controls, showed abnormal self-referential processing in both early perception and higher cognitive processing phases during the Self-Referential Encoding Task.

In “Dihydromyricetin alleviates diabetic neuropathic pain and depression comorbidity symptoms by inhibiting P2X7 receptor”, Guan et al. examined the effect of P2X7 receptor expression in the dorsal root ganglia (DRG), spinal cord, and hippocampus participates in the transduction of major depressive disorder. They found that dihydromyricetin can relieve MDD by reducing the expression of P2X7 receptor in the DRGs, spinal cord, and hippocampus and may be an effective new drug for the treatment of patients with both MDD.

In “An improved model of physical and emotional social defeat: different effects on social behavior and body weight of adolescent mice by interaction with social support”, Li et al. found that experiencing or witnessing a traumatic event during adolescence can increase the risk of psychiatric disorders in later lives.

In “Evidence-based dyadic therapies for 0-5 year old children with emotional and behavioral difficulties”, Romanowicz et al. reviewed four recommend child psychiatry psychotherapies for children with psychiatric and behavioral issues: Child-Parent Psychotherapy (CPP) and Trauma-Focused Cognitive Behavioral Therapy (TF-CBT), used primarily for young children with trauma, and Parent-Child Interaction Therapy (PCIT) and Child Parent Relationship Therapy (CPRT).

Taken together, the studies in this group strengthen the association between early developmental stress and trauma as being associated with the later development of depressive disorders. While the likely mechanisms underlying early development stress are likely multifactorial (involving genetic, physiological, and psychosocial disruptions, often in concert), attention to optimization of childhood environments and social experiences may result in some mitigation of risk of adult-onset depressive disorders. Given the ubiquity of MDD in the adult population, particularly among women, continued studies into early makers for risk of later depressive disorders remain a scientific, therapeutic, and public health imperative. As depressive disorder is a complex illness requiring comprehensive treatment, the research base to understand depressive disorder risk is necessarily complex and multifocal as well.

## Author Contributions

All authors were involved in the writing of the article.

## Conflict of Interest

The authors declare that the research was conducted in the absence of any commercial or financial relationships that could be construed as a potential conflict of interest.

